# Infant discrimination of humanoid robots

**DOI:** 10.3389/fpsyg.2015.01397

**Published:** 2015-09-22

**Authors:** Goh Matsuda, Hiroshi Ishiguro, Kazuo Hiraki

**Affiliations:** ^1^Department of Medical Education and General Medicine, Kyoto Prefectural University of MedicineKyoto, Japan; ^2^Department of General Systems Studies, Graduate School of Arts and Sciences, The University of TokyoTokyo, Japan; ^3^Department of Systems Innovation, Graduate School of Engineering Science, Osaka UniversityOsaka, Japan; ^4^Hiroshi Ishiguro Laboratories, Advanced Telecommunications Research Institute InternationalKyoto, Japan; ^5^CREST – Japan Science and Technology AgencyTokyo, Japan

**Keywords:** infant, humanoid robot, android, preferential looking paradigm, eye tracking, uncanny valley

## Abstract

Recently, extremely humanlike robots called “androids” have been developed, some of which are already being used in the field of entertainment. In the context of psychological studies, androids are expected to be used in the future as fully controllable human stimuli to investigate human nature. In this study, we used an android to examine infant discrimination ability between human beings and non-human agents. Participants (*N* = 42 infants) were assigned to three groups based on their age, i.e., 6- to 8-month-olds, 9- to 11-month-olds, and 12- to 14-month-olds, and took part in a preferential looking paradigm. Of three types of agents involved in the paradigm—a human, an android modeled on the human, and a mechanical-looking robot made from the android—two at a time were presented side-by-side as they performed a grasping action. Infants’ looking behavior was measured using an eye tracking system, and the amount of time spent focusing on each of three areas of interest (face, goal, and body) was analyzed. Results showed that all age groups predominantly looked at the robot and at the face area, and that infants aged over 9 months watched the goal area for longer than the body area. There was no difference in looking times and areas focused on between the human and the android. These findings suggest that 6- to 14-month-olds are unable to discriminate between the human and the android, although they can distinguish the mechanical robot from the human.

## Introduction

Over the last decade, various types of humanoid robots have emerged beyond the hypothetical realm of science fiction and into real life. More recently, robots with an extremely humanlike appearance, called “androids,” were developed (Ishiguro, 2006), primarily for interaction with humans. Because the best communicative partner of human beings is undoubtedly other humans, the development of a more humanlike appearance and motion for robots is considered a shortcut to developing robots that will have natural interactions with humans. Thus, investigating how currently available robots are perceived by humans will provide valuable information for this purpose.

The famous “uncanny valley” hypothesis is related to the impression conveyed by robots and their human likeness (Mori, 1970, 2012), and states that extremely humanlike artifacts often elicit negative affect, e.g., a feeling of eeriness, whereas modestly humanlike artifacts evoke familiarity. It was originally a theoretical hypothesis and remains controversial (Burleigh et al., 2013); some subsequent studies have, however, found empirical evidence supporting the existence of a similar phenomenon in both humans (Seyama and Nagayama, 2007) and other primates (Steckenfinger and Ghazanfar, 2009). In other words, the uncanny valley hypothesis suggests that humans have a sophisticated ability to discriminate between human and non-human beings. In fact, it has been reported that 80% of adult participants recognized that an android with a highly humanlike appearance was not a real human within 1 s (Noma et al., 2006), and that brain activity when viewing a human vs. an android is significantly different, especially in the anterior intraparietal sulcus, which is involved in action perception (Saygin et al., 2011). Currently available androids, therefore, do not seem to have achieved a sufficiently humanlike appearance in the view of human adults.

On the other hand, little is known about infant perception of extremely humanlike artifacts, such as androids. Newborns show primary discrimination abilities in relation to human properties, such as faces, voices, and movements (Goren et al., 1975; DeCasper and Fifer, 1980; Moon et al., 1993; Simion et al., 2008), and gradually gain more expertise during the first year of life. For example, whereas newborns can discriminate their mothers from strangers when the mothers’ heads are uncovered (Bushneil et al., 1989), they cannot do so when both women are wearing head scarves (Pascalis et al., 1995), although this only occurs up to 5 weeks of age (Bartrip et al., 2001). Moreover, at around 7 months, infants become able to process detailed facial configurations, such as the distance between eyes and mouth (Cohen and Cashon, 2001), and to identify strangers’ faces from a non-frontal view (Fagan, 1976). Discrimination of biological (e.g., a walking hen) from non-biological motion has also been observed in newborns (Simion et al., 2008), but the ability to differentiate human motion (e.g., a walking person) from non-human motion appears around 3 months of age (Bertenthal et al., 1987). By around 12 months of age, infants are able to discriminate possible and impossible human movements, such as fingers or elbows bending in the opposite direction (Christie and Slaughter, 2010; Morita et al., 2012). As mentioned above, although young infants already have primary discrimination abilities in relation to humans, this is not as well-developed as it is in adults. Therefore, it is likely that infant perception of humanoid robots is different from that of adults.

Investigating infant perception of androids inevitably leads to manifesting how infants discriminate human beings from non-human beings. Androids can be regarded as a highly controlled human stimuli for use in investigating human nature in the field of cognitive science (MacDorman and Ishiguro, 2006). Some researchers have already used androids as experimental stimuli (Saygin et al., 2011; Urgen et al., 2013); however, most targeted human adults. To our knowledge, there is only one study in which preschoolers’ responses to a real human and an android were compared (Moriguchi et al., 2010), and no studies on younger infants. Therefore, the purpose of this study was to investigate infant discrimination ability in regard to human beings, using humanoid robots and the preferential looking paradigm. When two kinds of stimuli are presented simultaneously in front of infants, a remarkable difference in looking times between both stimuli indicates that infants can discriminate between each stimulus. This method was devised by Fantz in the 1950s (Fantz, 1958), and is still widely used today in the field of developmental science.

In this study, three agents—a human, an android modeled on the human, and a mechanical-looking robot made from the android—were used as the experimental stimuli. If infants can recognize relatively few differences between the human and the android, significant difference in their looking times to each agent should be observed. Taking the findings of previous studies described above into consideration, it is very likely that younger infants will not realize that the android is not a human, while infants aged over 12 months may be able to discriminate between the two; therefore, this study targeted infants aged between 6 and 14 months. Furthermore, we employed an eye tracking system to measure infant looking times because it allows for more objective measurement and more precise analysis of focused areas than manual coding does. Even if no difference is found in looking times, there may be difference in the regions infants focus on when looking at each agent. Thus, this study will provide new evidence in relation to infants’ ability to discriminate human beings from non-human beings, and the pathway by which this ability develops. In addition, from the viewpoint of robotics, this experiment will evaluate the infant’s perception of the human likeness of currently available androids. If the uncanny valley hypothesis applies in infancy, particular responses to the android, such as avoiding viewing the android, may be observed.

## Materials and Methods

### Participants

Infants (*N* = 42; 20 boys, 22 girls; age = 6–14 months) were assigned to three groups based on their age: 6–8 months (six boys, five girls, mean age = 223.73 days, *SD* = 20.39), 9–11 months (eight boys, nine girls, mean age = 291.63 days, *SD* = 30.63), and 12–14 months (six boys, eight girls, mean age = 355.39 days, *SD* = 64.43). A further 22 infants were excluded from analysis following cessation of the experiment due to fussiness, such as crying and inability to stay still (*n* = 7), or a lack of valid gaze data (*n* = 15). Details about the criteria for data exclusion are described in the data analysis subsection below.

This study was approved by the ethics committee of the University of Tokyo. Written informed consent was obtained from the parents of all participants before beginning the experiment.

### Stimuli and Apparatus

**FIGURE 1:**
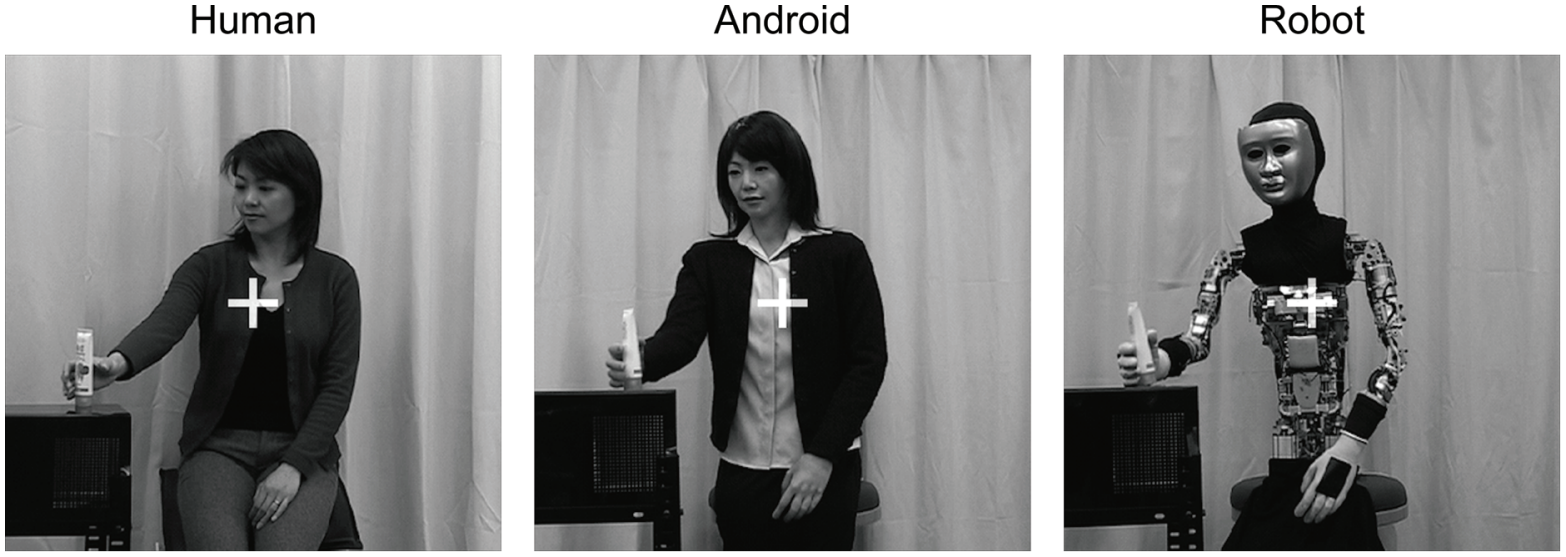
**Agents used as experimental stimuli.** The android was designed to have the likeness of the human actor, and was identical in internal architecture to the robot. The original face of the robot was covered with a plastic mask to conceal its somewhat bizarre appearance, with naked eyeballs and gums.

The visual stimuli were three different black and white video clips (800 × 800 pixels, 30 fps) that depicted one of three agents (a human, an android, or a mechanical robot) performing a grasping action with their right hand. **Figure 1** shows example frames of each video clip. These clips were made from stimuli used in a previous study (Saygin et al., 2011).

In the human agent clip, a Japanese woman reached her right hand toward a tube of facial wash, grasped it for a moment, and then moved her hand back to the original position. Her facial expression did not change and her left hand remained on her left thigh. In the android and robot clips, a female android named Repliee Q2 (Osaka University and KOKORO Co. Ltd., Japan) and a mechanical humanoid robot, respectively, performed the same grasping action as the human stimulus. The Repliee Q2 was modeled on the women actor shown in the human stimulus, and its upper body is moved by air actuators. Because the mechanical robot was made by stripping away the clothing and silicone skin from the android, the robots were almost identical in terms of physical size and motion. Although the robots’ motions were programed to resemble the human’s action as much as possible, those were actually rather unnatural due to mechanical limitations. In more concrete terms, whereas the human moved her hand straight to the target, the robots moved their hands over the target and then down toward it. All of the video clips were 3.5 s in duration, the second half (1.75 s) of which consisted of the first half (1.75 s) being played backwards. In addition, we used a simple animation with cheerful music that depicts a star changing in color and size as an attention getter.

Gaze data were collected at 300 Hz by the Tobii TX300 (Tobii AB, Sweden) contactless eye tracking system, which was placed at the center of a table. Its back and left and right sides were surrounded with curtains to ensure that the infants’ concentration remained on the stimuli. The stimuli were presented on a 23 in liquid crystal display (1920 × 1080 pixels) integrated with the Tobii, and the actual size of each video clip on the display was a 21 cm square. A small video camera (CCD-MC100, Sony Corporation) was additionally attached at the center of the upper frame of the display so that we could observe participants’ behavior. During gaze measurement, an experimenter who was located in an area separated by the curtain manipulated the Tobii and the stimuli.

### Procedure

Infants viewed the stimuli while sitting on their parent’s lap, and the distance between the infants and the display was approximately 60 cm. The tilt angle of the Tobii was adjusted so that it only captured infants’ eyes, and then a 5-point calibration was conducted. The parent was instructed not to respond to either the infant or the stimuli. In a single trial, two different video clips were presented at the same time side-by-side on the display, and were repeated three times without an interval. Thus, a single trial lasted 10.5 s. Each pair of agents (human vs. android: HA, human vs. robot: HR, and android vs. robot: AR) was presented four times, and the distance between two clips was 3.2 cm. The position (left or right) of the stimuli was counterbalanced. We conducted 12 trials if the infant did not become fussy, with the presentation order of each pair randomized. Before every trial, the attention getter was played at the center of the display until the infant looked toward it. Validity of eye tracking was monitored in real time using the “Show Track Status” function of the Tobii. An experimenter determined termination of the attention getter based on this status monitor and live footage from the video camera. In addition, the experimenter asked parents to move infants back to the initial position after a trial in which the Tobii lost infants’ eye gaze because they moved vigorously.

### Analysis

Trials with invalid (missing) gaze data for more than 50% of the trial duration were excluded from the data analysis. Moreover, participants for whom the data of one or more agent pairs was not obtained at all, were completed excluded. There were 15 infants excluded based on this criterion, primarily due to a hardware failure of the Tobii TX300 eye tracking system. According to the developer of Tobii, when the TX300 is used with a particular firmware (ver. 1.1.0), as we did in this study, it can fail to detect infant gaze during high-frequency measurement because of a problem in its algorithm for gaze detection. This problem does not occur in measurement at lower frequencies, such as at 60 and 120 Hz, and it has been fixed in the latest firmware (ver. 1.1.1). Regrettably, we lost a large amount of data because we were not aware of this important problem and its solution until after the experiment was complete.

**FIGURE 2:**
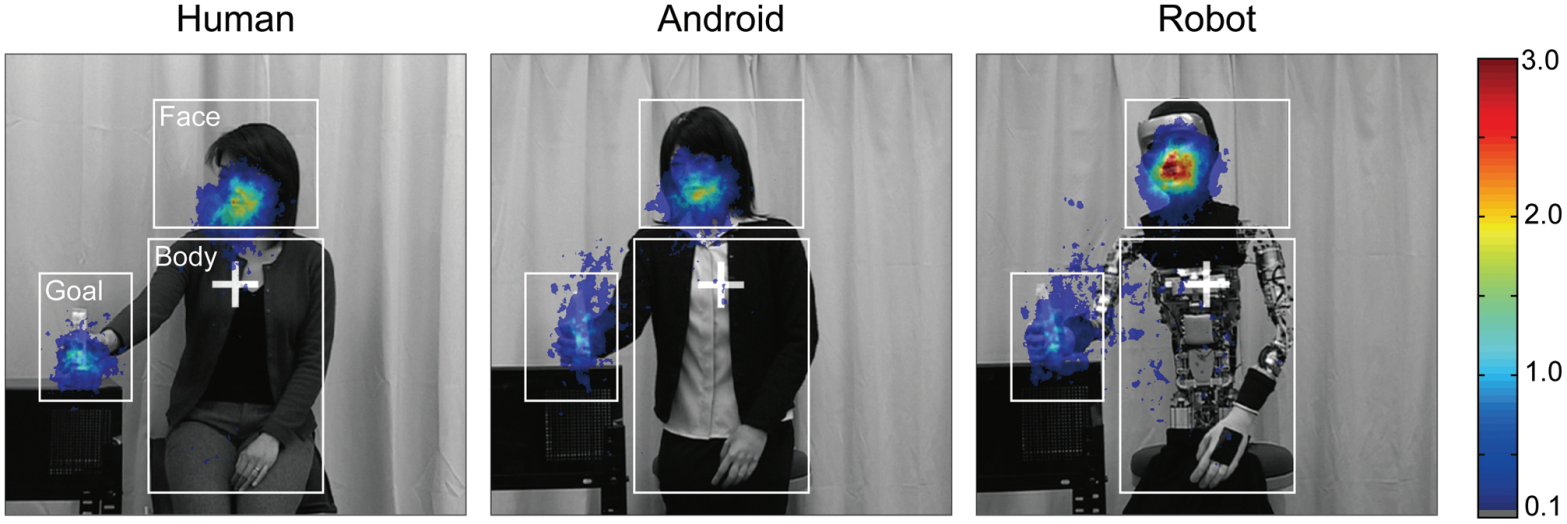
**Heat maps of mean gaze count across all trials of all participants, superimposed upon each agent after 7 × 7 pixel Gaussian smoothing was applied.** Red represents an area that the greatest number of infants viewed. areas of interest (AOI) are depicted as white rectangles. The reason for the focused areas in the goal area of the android and the robot spreading vertically is probably due to the trajectories of the agents’ hands.

We defined three static areas of interest (AOI), corresponding to the face area, a goal area, and the body area (see **Figure 2**). The same three AOI were applied to each agent, and statistical analysis was performed separately for each pair of agents (HA, HR, and AR). To calculate the proportions of looking times toward each AOI of each agent, mean gaze counts were divided by the total gaze count for two agents presented simultaneously. One gaze count corresponds to 3.3 ms viewing at 300 Hz sampling. A three-way mixed design analysis of variance (ANOVA; age group × agent × AOI) with the arcsine transformation was conducted for the proportions of looking times, and the Huynh–Feldt correction for degrees of freedom was employed as necessary. Multiple comparison with the Bonferroni method was carried out when an interaction was found.

## Results

To make it easier to understand the overall trends, heat maps of the mean gaze count across all trials of all participants for each agent are shown in **Figure 2**.

**FIGURE 3:**
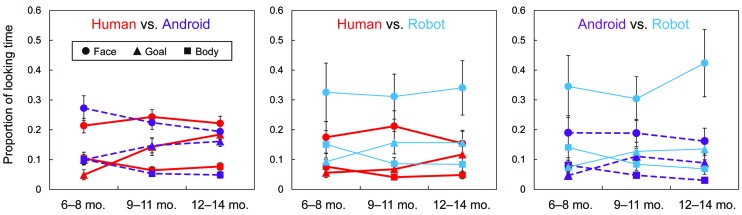
**Proportions of total looking times at each AOI of each agent across the three age groups.** Red solid lines, purple dotted lines, and blue thin lines represent the human, android, and robot agents, respectively. Circle, triangle, and square markers correspond to AOI of face, goal, and body, respectively. Error bars represent standard errors.

**Figure 3** shows the mean proportions of looking time for each AOI in each age group. There were main effects of agent in the HR and AR conditions [HR: *F*(1,39) = 22.65, *p* < 0.001; AR: *F*(1,39) = 28.90, *p* < 0.001], of AOI in all three conditions [HA: *F*(1.83, 71.29) = 45.86, *p* < 0.001; HR: *F*(1.70, 66.23) = 50.64, *p* < 0.001; AR: *F*(1.67, 65.29) = 64.46, *p* < 0.001), and of age group only in the HA condition [*F*(2,39) = 5.83, *p* = 0.006].

Moreover, an interaction between age group and AOI was found in all of the three conditions [HA: *F*(3.66, 71.29) = 5.19, *p* = 0.001; HR: *F*(3.40, 66.23) = 2.77, *p* < 0.05; AR: *F*(3.35, 65.29) = 3.17, *p* < 0.05]. The details of significant differences between each AOI in each age group and those between each age group at each AOI are described in **Tables 1** and **2** respectively. **Table 1** shows that infants in all age groups principally watched the face area of each agent, and that infants aged over 9 months watched the goal area for longer than they did the body area. Further, **Table 2** shows the gaze preference for the goal area in infants aged over 9 months, and shows that the 6- to 8-month-old group tended to view the body area for longer than the older groups did.

**Table 1:** The results of multiple comparisons for looking times between each areas of interest (AOI; Face/Goal/Body) in each age group (6–8/9–11/12–14 months).

Age (months)	Human vs. Android	*p*<	Human vs. Robot	*p*<	Android vs. Robot	*p*<
6–8	Face > Goal	0.001	Face > Goal	0.001	Face > Goal	0.001
	Face > Body	0.01	Face > Body	0.01	Face > Body	0.001
			Body > Goal	0.05		
9–11	Face > Goal	0.05	Face > Goal	0.001	Face > Goal	0.01
	Face > Body	0.001	Face > Body	0.001	Face > Body	0.001
	Goal > Body	0.001	Goal > Body	0.05		
12–14	Face > Body	0.001	Face > Goal	0.05	Face > Goal	0.001
	Goal > Body	0.001	Face > Body	0.001	Face > Body	0.001
			Goal > Body	0.01	Goal > Body	0.05

*Mean values and standard errors are represented in **Figure 3**, while significant differences are described in this table. An inequality of “A > B” means that the looking time toward the AOI of A was significantly longer than that toward the AOI of B.*

**Table 2:** The results of multiple comparisons for looking times between each age group (6–8/9–11/12–14 months) in each AOI (Face/Goal/Body).

AOI	Human vs. Android	*p*<	Human vs. Robot	*p*<	Android vs. Robot	*p*<
Face	n.s.		n.s.		n.s.	
Goal	9–11 > 6–8	0.01	12–14 > 6–8	0.05		
	12–14 > 6–8	0.001			n.s.	
Body	6–8 > 9–11	0.05	6–8 > 9–11	0.05	6–8 > 9–11	0.05
					6–8 > 12–14	0.01

*All mean values and standard errors are represented in **Figure 3**, while significant differences are described in this table. An inequality of “A > B” means that the looking time of group A was significantly longer than that of group B.*

An interaction of agent and AOI was also found in the HR and AR conditions [HR: *F*(2,78) = 3.53, *p* < 0.05; AR: *F*(2,78) = 12.53, *p* < 0.001]. Multiple comparison revealed that the robot captured the longest looking time among all of the agents in any AOI (*p* < 0.05 for the goal area in the AR condition, *p* < 0.01 for the goal area in the HR condition and for the body area in the AR condition, *p* < 0.001 for the rest), and that infants viewed the face area for significantly longer than they did the other AOI (all *p*s < 0.001).

No second-order interactions were found in any conditions. Further, no effect and interaction involved in the agent factor was detected in the HA condition; that is, there were no significant differences in either looking time or focusing area between the human and the android in any age groups.

## Discussion

To examine infant discrimination ability among human and humanlike agents and to test the human likeness of a currently available android, we measured looking times of infants aged between 6 and 14 months in regard to three types of agents of similar body size and motion. The three-way ANOVA revealed that infants of all age groups spent the longest time on viewing the robot, especially its face, compared with the other agents. Further, there was no difference in looking time between the human and android agents. These results suggest that 6- to 14-month-old infants are unable to distinguish the android from the human, although they are able to distinguish the robot from the human.

Infants’ gaze preference for the mechanical robot is probably derived from their novelty preference tendency. A considerable number of studies have shown that infants generally prefer unfamiliar to familiar stimuli. The fact that the preference was observed in the AR condition, where the motions of both agents were almost the same, indicates that the visual aspects of the robot, rather than the motion, captured the infants’ attention. Although it is likely that the infants who participated in our experiment often saw many women besides their mother in daily life, none had seen the robot before taking part in this study; therefore, the robot must have been the most unfamiliar to them from among the three agents.

Despite the fact that the android is also a rare stimulus for the infants to have observed in reality, there was no gaze preference between the human and android agent. An absence of preference for the looking paradigm does not directly indicate that two stimuli are considered to be identical; hence, it is unclear whether the infants regarded the human and the android as the same person. However, our findings suggest, at least, that the human and the android were regarded as equally humanlike beings.

A similar insensitivity to artificial humanity in infants has been reported by a previous study (Lewkowicz and Ghazanfar, 2012), where it was exhibited that 6- to 12-month-old infants were unable to discriminate a realistic computer graphics (CG) avatar from a real human. Although the authors used the term “realistic” to describe their stimuli, the stimuli actually had a non-photorealistic appearance that any adult could recognize as being a CG avatar at a glance. Our android had a more photorealistic appearance than theirs did; therefore, it should have been difficult for not only 6- to 12-month-old infants but also older infants to discriminate between the human and the android.

The motion of the android used in this study was unnatural due to its mechanical limitations. If infants recognize the unnaturalness of its motion, it is possible that they looked for longer at the android than at the human; however, the results showed that this was not the case. The android’s grasping action is somewhat awkward but not impossible for human beings. It is likely that the discrimination ability of infants aged around 1 year for human movement is not yet sophisticated enough to detect this type of awkwardness.

In all the three conditions and for all age groups, infants spent the longest time looking at the face AOI. Infants’ preference for faces has been reported by many previous researchers. Even newborns under 1 week of age prefer face and face-like stimuli to other stimuli (Goren et al., 1975; Macchi et al., 2004; Farroni et al., 2005), and infants gradually focus their attention on faces at between 3 and 9 months of age (Frank et al., 2009). This preference for faces has been observed regardless of the nature of the stimuli, i.e., geometric or photographic images (Farroni et al., 2005), and is, thus, considered to reflect the importance of faces in human communication (Csibra and Gergely, 2009).

Interestingly, looking times in the goal AOI were larger in the older infant groups than in the youngest group. This probably depends on the development of their prediction ability for human action. Falck-Ytter et al. (2011) compared looking behaviors of 6- and 12-month-old infants and adults while watching human goal-directed actions, and revealed that 12-month-olds and adults looked at the goal area significantly faster and for longer than 6-month-olds did. In another similar study (Kanakogi and Itakura, 2011), the authors proposed that this prediction ability for others’ actions corresponds to their own motor ability, and demonstrated that infant grasping ability develops gradually after 6 months of age. Our result is highly consistent with these findings. A shorter looking at the goal AOI in the 6- to 8-month-old group may reflect their rudimentary understanding of the goal of the agents’ action.

Of course, there are limitations in our study. First, it is possible that the stimuli were too small for infants to detect slight differences in appearance and motion between the human and the android. We used 21 cm square black and white video clips, which were presented 60 cm away from the infants. An agent of this size corresponds to a real agent at about 2.5 m distance. The presentation of a real android may produce different results. In fact, presentation at a realistic size facilitates information processing about the human body in young infants (Heron and Slaughter, 2010). Second, factors that can influence the perceived human likeness of robots are not limited to their appearance and motion. For example, a study using a mechanical humanoid robot reported that infants regarded the robot as a communicative agent only after watching interactions between a human and the robot (Arita et al., 2005). This finding implies that the interactive functions of robots can influence their human likeness. In addition, infants’ characteristics, such as gender, and temperament, influence the perceived human likeness of robots. Because female, compared to male, infants have been reported to show an advantage in processing social stimuli, such as facial expressions (McClure, 2000), and to prefer more human-like stimuli, such as dolls and human faces (Connellan et al., 2000; Lutchmaya and Baron-Cohen, 2002; Alexander et al., 2009), their ability to discriminate between human and non-human beings may mature faster. Finally, gaze measurement is not the only way to investigate infant discrimination ability. Recently, infants’ neural response to stimuli has been attracting attention as a new subjective index of their discrimination ability, in association with the development of non-invasive and more simplified technology for measuring brain activity (Csibra et al., 2004; Farroni et al., 2004). Although we did not find differences in infant gaze behaviors between the human and the android agents in this study, infants’ neural response to the two types of agent may differ in some brain regions.

To our knowledge, this is the first report concerning infant discrimination of a recently developed android from humans and robots. Our results suggest that discrimination ability in regard to human vs. non-human beings is not as sophisticated in infants younger than 14 months as it is in adults. The uncanny valley effect elicited by the android was not found in infants; in other words, a currently available android may have already reached a humanlike quality for infants, at least with regard to appearance and motion. Androids have great potential as an alternative to human stimuli in future psychological studies.

## Conflict of Interest Statement

The authors declare that the research was conducted in the absence of any commercial or financial relationships that could be construed as a potential conflict of interest.

## References

[B1] Alexander G. M. Wilcox T. Woods R. (2009). Sex differences in infants’ visual interest in toys. *Arch. Sex. Behav.* 38 427–433. 19016318 10.1007/s10508-008-9430-1

[B2] Arita A. Hiraki K. Kanda T. Ishiguro H. (2005). Can we talk to robots? Ten-month-old infants expected interactive humanoid robots to be talked to by persons. *Cognition* 95 B49–B57. 10.1016/j.cognition.2004.08.001 15788157

[B3] Bartrip J. Morton J. Schonen S. (2001). Responses to mother’s face in 3-week to 5-month-old infants. *Br. J. Dev. Psychol.* 19 219–232. 10.1348/026151001166047

[B4] Bertenthal B. I. Proffitt D. R. Kramer S. J. (1987). Perception of biomechanical motions by infants: implementation of various processing constraints. *J. Exp. Psychol. Hum. Percept. Perform.* 13 577–585. 10.1037/0096-1523.13.4.577 2965749

[B5] Burleigh T. J. Schoenherr J. R. Lacroix G. L. (2013). Does the uncanny valley exist? An empirical test of the relationship between eeriness and the human likeness of digitally created faces. *Comput. Hum. Behav.* 29 759–771. 10.1016/j.chb.2012.11.021

[B6] Bushneil I. Sai F. Mullin J. (1989). Neonatal recognition of the mother’s face. *Br. J. Dev. Psychol.* 7 3–15. 10.1111/j.2044-835X.1989.tb00784.x

[B7] Christie T. Slaughter V. (2010). Movement contributes to infants’ recognition of the human form. *Cognition* 114 329–337. 10.1016/j.cognition.2009.10.004 19889403

[B8] Cohen L. B. Cashon C. H. (2001). Do 7-month-old infants process independent features or facial configurations? *Infant Child Dev.* 10 83–92. 10.1002/icd.250

[B9] Connellan J. Baron-Cohen S. Wheelwright S. Batki A. Ahluwalia J. (2000). Sex differences in human neonatal social perception. *Infant Behav. Dev.* 23 113–118. 10.1016/S0163-6383(00)00032-1

[B10] Csibra G. Gergely G. (2009). Natural pedagogy. *Trends Cogn. Sci.* 13 148–153. 10.1016/j.tics.2009.01.005 19285912

[B11] Csibra G. Henty J. Volein A. Elwell C. Tucker L. Meek J. (2004). Near infrared spectroscopy reveals neural activation during face perception in infants and adults. *J. Pediatr. Neurol.* 2 85–90.

[B12] DeCasper A. J. Fifer W. P. (1980). Of human bonding: newborns prefer their mothers’ voices. *Science* 208 1174–1176. 10.1126/science.7375928 7375928

[B13] Fagan J. F. III. (1976). Infants’ recognition of invariant features of faces. *Child Dev.* 47 627–638. 10.1111/j.1467-8624.1976.tb02226.x

[B14] Falck-Ytter T. Bakker M. Von Hofsten C. (2011). Human infants orient to biological motion rather than audiovisual synchrony. *Neuropsychologia* 49 2131–2135. 10.1016/j.neuropsychologia.2011.03.040 21477604

[B15] Fantz R. L. (1958). Pattern vision in young infants. *Psychol. Rec.* 8 43–47.

[B16] Farroni T. Johnson M. H. Csibra G. (2004). Mechanisms of eye gaze perception during infancy. *J. Cogn. Neurosci.* 16 1320–1326. 10.1162/0898929042304787 15509381

[B17] Farroni T. Johnson M. H. Menon E. Zulian L. Faraguna D. Csibra G. (2005). Newborns’ preference for face-relevant stimuli: effects of contrast polarity. *Proc. Natl. Acad. Sci. U.S.A.* 102 17245–17250. 10.1073/pnas.0502205102 16284255 PMC1287965

[B18] Frank M. C. Vul E. Johnson S. P. (2009). Development of infants’ attention to faces during the first year. *Cognition* 110 160–170. 10.1016/j.cognition.2008.11.010 19114280 PMC2663531

[B19] Goren C. C. Sarty M. Wu P. Y. (1975). Visual following and pattern discrimination of face-like stimuli by newborn infants. *Pediatrics* 56 544–549. 1165958

[B20] Heron M. Slaughter V. (2010). Infants’ responses to real humans and representations of humans. *Int. J. Behav. Dev.* 34 34–45. 10.1177/0165025409345047

[B21] Ishiguro H. (2006). Android science: conscious and subconscious recognition. *Conn. Sci.* 18 319–332. 10.1080/09540090600873953

[B22] Kanakogi Y. Itakura S. (2011). Developmental correspondence between action prediction and motor ability in early infancy. *Nat. Commun.* 2 341 10.1038/ncomms1342 21654641

[B23] Lewkowicz D. J. Ghazanfar A. A. (2012). The development of the uncanny valley in infants. *Dev. Psychobiol.* 54 124–132. 10.1002/dev.20583 21761407 PMC3197970

[B24] Lutchmaya S. Baron-Cohen S. (2002). Human sex differences in social and non-social looking preferences, at 12 months of age. *Infant Behav. Dev.* 25 319–325. 10.1016/S0163-6383(02)00095-4

[B25] Macchi C. V. Turati C. Simion F. (2004). Can a nonspecific bias toward top-heavy patterns explain newborns’ face preference? *Psychol. Sci.* 15 379–383. 10.1111/j.0956-7976.2004.00688.x 15147490

[B26] MacDorman K. F. Ishiguro H. (2006). The uncanny advantage of using androids in cognitive and social science research. *Interact. Stud.* 7 297–337. 10.1075/is.7.3.03mac

[B27] McClure E. B. (2000). A meta-analytic review of sex differences in facial expression processing and their development in infants, children, and adolescents. *Psychol. Bull.* 126 424–453. 10.1037/0033-2909.126.3.424 10825784

[B28] Moon C. Cooper R. P. Fifer W. P. (1993). Two-day-olds prefer their native language. *Infant Behav. Dev.* 16 495–500. 10.1016/0163-6383(93)80007-U

[B29] Mori M. (1970). Bukimi no tani [The uncanny valley]. *Energy* 7 33–35.

[B30] Mori M. (2012). The uncanny valley. *IEEE Robot. Autom. Mag.* 19 98–100. 10.1109/Mra.2012.2192811

[B31] Moriguchi Y. Minato T. Ishiguro H. Shinohara I. Itakura S. (2010). Cues that trigger social transmission of disinhibition in young children. *J. Exp. Child Psychol.* 107 181–187. 10.1016/j.jecp.2010.04.018 20547394

[B32] Morita T. Slaughter V. Katayama N. Kitazaki M. Kakigi R. Itakura S. (2012). Infant and adult perceptions of possible and impossible body movements: an eye-tracking study. *J. Exp. Child Psychol.* 113 401–414. 10.1016/j.jecp.2012.07.003 22906302

[B33] Noma M. Saiwaki N. Itakura S. Ishiguro H. (2006). “Composition and evaluation of the humanlike motions of an android,” in *Proceedings of the 2006 6th IEEE-RAS International Conference on Humanoid Robots* (Genova: IEEE), 163–168. 10.1109/ichr.2006.321379

[B34] Pascalis O. De Schonen S. Morton J. Deruelle C. Fabre-Grenet M. (1995). Mother’s face recognition by neonates: a replication and an extension. *Infant Behav. Dev.* 18 79–85. 10.1016/0163-6383(95)90009-8

[B35] Saygin A. P. Chaminade T. Ishiguro H. Driver J. Frith C. (2011). The thing that should not be: predictive coding and the uncanny valley in perceiving human and humanoid robot actions. *Soc. Cogn. Affect. Neurosci.* 7 413–422. 10.1093/scan/nsr025 21515639 PMC3324571

[B36] Seyama J. Nagayama R. S. (2007). The Uncanny Valley: effect of realism on the impression of artificial human faces. *Presence* 16 337–351. 10.1162/pres.16.4.337

[B37] Simion F. Regolin L. Bulf H. (2008). A predisposition for biological motion in the newborn baby. *Proc. Natl. Acad. Sci. U.S.A.* 105 809–813. 10.1073/pnas.0707021105 18174333 PMC2206618

[B38] Steckenfinger S. A. Ghazanfar A. A. (2009). Monkey visual behavior falls into the uncanny valley. *Proc. Natl. Acad. Sci. U.S.A.* 106 18362–18366. 10.1073/pnas.0910063106 19822765 PMC2760490

[B39] Urgen B. A. Plank M. Ishiguro H. Poizner H. Saygin A. P. (2013). EEG theta and Mu oscillations during perception of human and robot actions. *Front. Neurorobot.* 7:19 10.3389/fnbot.2013.00019 PMC3826547 24348375

